# Effect of Raw Material, Pressing and Glycosidase on the Volatile Compound Composition of Wine Made From Goji Berries

**DOI:** 10.3390/molecules21101324

**Published:** 2016-10-02

**Authors:** Guanshen Yuan, Jie Ren, Xiaoyu Ouyang, Liying Wang, Mengze Wang, Xiaodong Shen, Bolin Zhang, Baoqing Zhu

**Affiliations:** 1 Beijing Key Laboratory of Forestry Food Processing and Safety, Department of Food Science, College of Biological Sciences and Biotechnology, Beijing Forestry University, Beijing 100083, China; guanshenyuan@126.com (G.Y.); chxdyd2012@163.com (J.R); oyxy1993@sina.com (X.O.); zhangbolin888@163.com (B.Z); 2 Ningxia Senmiao Goji Technology Development Co. Ltd., Yinchuan, Ningxia 750000, China; wangliying@senmiao.com (L.W.); wangmengze@senmiao.com (M.W.); shenxiaodong@senmiao.com (X.S.)

**Keywords:** goji wine, dried goji berry, fresh goji berry, pressing, free and pressed juice, glycosidase, aromatic compounds

## Abstract

This study investigated the effect of raw material, pressing, and glycosidase on the aromatic profile of goji berry wine. The free-run and the pressed juice of dried and fresh goji berries were used for wine production, whereas glycosidase was applied to wine after fermentation. Dried goji berry fermented wine exhibited much stronger fruity, floral, caramel, and herbaceous odors due to higher levels of esters, β-ionone and methionol. However, fresh berry fermented wine possessed stronger chemical notes due to higher levels of 4-ethylphenol. Pressing treatment reduced the fruity and caramel odors in these fermented wines, and fresh berry free-run juice fermented wine exhibited the least floral aroma. Glycosidase addition did not alter the aromatic composition of wines. The principal component analysis indicated that goji raw material played a primary role in differentiating the aromatic profiles of the wines due to the difference on the content of 20 esters, nine benzenes, eight aldehydes/ketones, three acids, two alcohols and six other volatiles. The content differences on isopentyl alcohol, styrene, benzyl alcohol, 1-octanol, (*E*)-5-decen-1-ol, 1-hexanol, and β-cyclocitral resulted in the segregation of the wines with and without the pressing treatment, especially for fresh berry fermented wine.

## 1. Introduction

Goji (*Lycium barbarum* L.) is an important economic crop widely cultivated in the northwestern regions of China [1]. Goji berries are consumed as a functional food due to their multiple health properties [2]. They have been reported to have the capacity to decrease the incidence of vision, liver, kidney, and immune system problems because of their high levels of functional ingredients, such as polysaccharides, flavonoids and carotenoids [3,4,5]. 

Goji berries are indefinite inflorescences, and the fruits normally mature between June and August. Therefore, ripe goji berry fruits have to be harvested several times a year. Fresh goji berries readily spoil during storage, and therefore the majority (about 90%) of fresh goji berries are normally processed into dried products. Additionally, the goji berry wine fermentation industry has attracted attention in China, since goji berry wine can preserve the nutritional value of goji bery and extend the shelf-life [6].

The overall aroma determines the sensory feature and quality of any fruit wine. The overall aroma in fruit wine is comprised of the primary, secondary, and tertiary aromas [7]. The primary aroma consists of volatile compounds derived from the fruit [7]. Fermentation results in the production of volatile compounds with fermented notes [8]. The evolution of volatiles during the aging process can bring aging odors to wine [8]. Fruit-derived aromatic compounds essentially determine the typical flavor of fruit wine [9]. Several strategies have been applied in the wine industry to investigate their effect on the extraction of fruit-derived volatiles. For example, pressing treatment is a physical approach to disintegrate the structure of fruit cells, enhancing the release of aromatic compounds and volatile precursors in juice. It has been reported that the pressed juice contained higher levels of C_6_ alcohols compared to the free-run juice [10]. Besides, odorless volatile precursors exist in fruit with a large amount [9]. Glycosidase can cleave the sugar moieties from bound volatiles to release free volatiles that can contribute to the overall aroma of wine [9,11,12,13]. For example, terpene alcohols are the major volatile aglycones released from their precursors by glycosidase in grape wine, and these aromatic compounds can improve the floral and fruity notes of the overall aroma of grape wine [9,11,12,13].

The effect of pressing and glycosidase on the evolution of volatile composition during grape wine production has been well studied [9,14]. However, such investigations on goji berry wine production have barely been documented. More importantly, fresh goji berry is always processed into a dried form. The drying process can cause dramatic changes in the volatile composition and aromatic precursors in dried goji berry, which may alter the aromatic profile in goji berry wine. Therefore, we selected dried and fresh goji berries, and applied pressing and glycosidase treatments during the goji berry wine-making process. The objective of this study was to investigate the effect of goji berry raw material, pressing treatment, and glycosidase application on the volatile composition of goji berry wine, which could provide practical knowledge on quality development of goji berry wine fermentation. More importantly, the findings from this study could also provide a useful reference on material selection and fermentation processing development using fruits different from grapes. 

## 2. Results and Discussion

### 2.1. Physicochemical Parameters of Goji Wine

Alcohol strength, volatile acidity, titratable acidity, residual sugar, polysaccharide, free SO_2_, total SO_2_, and pH of these goji berry fruit wines are listed in Table 1. These goji berry fruit wines contained the high level of the titratable acidity and the pH value, indicating that these wines exhibited a stronger buffer capacity. This wine feature might be attributed to that goji berry is cultivated in a high saline level soil condition. 

Additionally, polysaccharide is an important bioactive component that is largely present in goji berry [15]. The level of polysaccharide in these goji berry wines ranged of 1–3 g/L, which was much higher than that in grape wines (0.2–1.5 g/L) [16]. Meanwhile, the dried goji berry wine made from pressed juice showed the polysaccharide content about 2 times higher than the other goji berry wines, whereas the fresh goji berry pressed juice wine did not show any increase on the level of polysaccharide. These indicated that the drying process of goji berry fruit might cause the structure change of goji berry fruit or polysaccharide in fruit, which resulted in such differences on the polysaccharide level in the wines.

These goji berry wines also showed high level of volatile acidity. It has been reported that yeasts under high osmotic stress condition generated more acetic acid in ice wine during fermentation [17]. We speculated that osmotic stress induced by high level polysaccharide in goji berry fruits might stimulate the yeasts to yield more volatile acids in the wine. However, a further investigation should be conducted.

The total SO_2_ content in these goji berry wines was much higher than that in goji berry fruits before the fermentation (Table 1). Potassium metabisulfite was added to terminate the fermentation process in this study, which resulted in the elevation of SO_2_ content in these wines. Meanwhile, the yeasts used in this study also possessed the ability of produce SO_2_ during the fermentation.

### 2.2. Volatile Composition in Wine Made of Dried and Fresh Goji Berry

The drying process has been reported to destroy the berry cell structure, resulting in the release of nutrients and enzymes [18,19,20,21]. These compounds further experience dehydrogenation, oxidation, degradation, and/or Maillard reactions, leading to the formation of a large number of volatiles [22]. It has been reported that the concentration of C_6_ alcohols and aldehydes, inducing the cut grass odor, significantly decreased in fruits during the drying process [23]. Meanwhile, some volatiles with spicy and ripe fruity notes were reported to be generated during the drying period, enhancing the odor complexity of dried fruit [23].

The volatile compounds identified in this work are listed in Table 2. A total of 97 volatile compounds were detected in the dried goji berry wine, whereas the fresh goji berry wine contained 94 volatile compounds (Table 3). Particularly, 2-acetylpyrrole, 3-phenyl-4-hydroxyacetophenone, isobutyl hexanoate, and cedrol were detected only in the dried goji berry wine, whereas *E*-5-decen-1-ol existed only in the fresh wine. In these wines, higher alcohols appeared to be the predominant volatiles in the goji berry wine, followed by esters, benzenes, and acids. However, aldehydes, ketones, terpenes, and norisoprenoids existed in these wines in low amounts. 

In this study, the fresh and the dried goji berry fermented wines showed similar volatile compositions. However, the concentration of these volatiles in the wine samples exhibited significant differences (Table 3 and Table 4). The fresh berry fermented wine showed higher total contents of aromatic compounds. A total of 56 volatiles displayed higher concentrations in the dried berry fermented wine, whereas the fresh goji berry fermented wine contained 31 volatiles that exhibited higher concentrations.

#### 2.2.1. Higher Alcohols

The higher alcohol content was significantly so in the fresh goji berry fermented wine (Table 3). Particularly, [*R*-(R*,R*)]- and [*S*-(R*,R*)]-2,3-butanediol, pentyl alcohol, 1-hexanol, 2-heptanol, 2-ethyl-1-hexanol, 1-octanol, 1-nonanol, 1-octen-3-ol, *trans*-2-octenol, and *E*-5-decen-1-ol showed a higher concentration in the fresh goji berry fermented wine, whereas the dried goji berry resulted in a wine with higher concentrations of 2-methyl-1-propanol and methionol. 2,3-Butanediol (including both the [*R*-(R*,R*)] and [*S*-(R*,R*)]) isomers) has been considered an important fermentation by-product that could alter the overall aroma of wine [24]. The obvious content differences in these wines might induce the different aromatic features. No significant differences on the content of 1-butanol, isopentyl alcohol, (*Z*)-3-hexen-1-ol, or 3-ethoxy-1-propanol were observed between the fresh and the dried goji berry fermented wines.

#### 2.2.2. Aldehydes and Ketones

Aldehydes and ketones are considered the important aromatic compounds in fruits, and the drying process has been reported to enhance their accumulation in dried fruits [22]. Furfural has been reported to be produced by the dehydration of sugar under acidic conditions, and its content increases as the drying period increases [25]. 2-Acetylpyrrole was synthesized from proline, hydroxyproline, and/or sugar under Strecker degradation conditions, and this volatile exhibits a burnt scent [26]. Decanal is derived from the oxidation of oleic acid in fruits during the drying process [27]. This volatile contributes soapy and green lemon odors to the overall aroma [28]. The dried goji berry fermented wine contained significantly higher concentrations of furfural, safranal, 2-butyl-2-octenal, isobutyl ketone, 6-methyl-5-heptene-2-one, 2-undecanone, β-cyclocitral, dihydropseudo-ionone, 2-acetylpyrrole, and (2,6,6-trimethyl-2-hydroxycyclohexylidene)acetic acid lactone (Table 3 and Table 4). Particularly, 6-methyl-5-heptene-2-one exists in a trace amounts in the fresh goji berry fermented wine, whereas the dried goji berry fermented wine contained this compound with much higher concentration. 6-Methyl-5-heptene-2-one is a product of the hydrolysis of carotenoids in fruits. We speculate that the drying process might result in the degradation of carotenoids, which leads to a higher concentration of 6-methyl-5-heptene-2-one in the dried goji berry fruit fermented wine.

#### 2.2.3. Volatile Acids

Volatile acids are produced by the metabolism of sugars, lipids, and amino acids during the wine fermentation process [29]. Although these fatty acids exist in wine in small amounts, they contribute significantly fatty, pungent, rancid, fruity, and cheesy odors to wines [30,31]. No difference in the acid composition was observed in the goji berry fermented wines produced from the different raw materials, however, their concentrations showed significant differences (Table 3 and Table 4). The dried goji berry fermented wine contained higher concentrations of 1,2-dimethyl-cyclopent-2-ene-carboxylic acid, butanoic acid, α-methylbutyric acid, and octanoic acid. Particularly, the concentration of 1,2-dimethylcyclopent-2-enecarboxylic acid in the dried goji berry fermented wine was 16 times higher than that in the fresh berry fermented wine. The higher level of butanoic acid in the dried berry fermented wine might result from its accumulation during the fruit postharvest application [32]. The fresh goji berry fermented wine showed three times higher concentration of acetic acid than the dried goji berry fermented wine. These wines had similar levels of decanoic acid.

#### 2.2.4. Esters

Esters are mainly formed as secondary products of yeast metabolism during the fermentation process [29]. Their levels in wine are mainly determined by those of their precursors in fruits [33]. It has been accepted that the drying process alters the composition of ester precursors in fruits [22] and as a result, a significant difference on the ester levels between fresh and the dried goji berry fermented wines might be expected. Ethyl acetate was the dominant ester in both goji wines, with a higher concentration in the dried berry fermented wine (Table 4). Similarly, the dried berry fermented wine also contained higher levels of the majority of the ester compounds, including 11 ethyl esters, six acetate esters, four fatty acid esters, and two other esters. Only six esters (ethyl 3-hexenoate, ethyl lactate, ethyl nonanoate, ethyl 2-hydroxy-4-methylpentanoate, ethyl hexadecanoate, and trimethylene acetate) exhibited higher concentrations in the fresh goji berry fermented wine. Both of the wines contained similar levels of ethyl myristate, isobutyl octanoate, and methyl caprate.

#### 2.2.5. Benzenes and Volatile Phenols

Although the levels of benzenes were not as high as those of alcohols and esters in the goji berry fermented wines, a significant difference on their concentration was also observed (Table 4). The fresh goji berry fermented wine contained higher concentrations of methyl 2-(pentyloxy) benzoate, ethyl dihydrocinnamate, coumaran, 4-ethylphenol and 4-vinylguaiacol. On average, the concentrations of 4-ethylphenol and 4-vinylguaiacol were 1474 μg/L and 346 μg/L in the dried berry fermented wine, which corresponds to only about 16% and 54% of that in the fresh berry fermented wine, respectively. Considering the differences of their amount between these wines, we speculated that the drying process decreased the level of their precursors in the goji berry fruits [31,32,33,34]. Besides, the drying process also reduced the population of microorganisms, such as *Dekkera bruxellensis*, on the surface of goji berry fruits. These microorganisms have also been reported to convert *p*-coumaric and ferulic acid into 4-ethylphenol and 4-vinylguaiacol during the fermentation process [34,35]. The dried goji berry fermented wine possessed higher concentrations of isodurene, 1,1,4,6-tetramethylindane, 2,5-diisopropyl-*p*-xylene, naphthalene, 2,5,8-trimethyl-1,2-dihydronaphthalene, phenethyl acetate, β-methylnaphthalene, α-methylnaphthalene, and 4-tolylcarbinol although these volatiles were all detected at low levels. Both the fresh and the dried goji berry fermented wines contained similar concentrations of ethyl cinnamate, 2,4-di-tert-butylphenol, and ethyl salicylate.

#### 2.2.6. Terpenes and Norisoprenoids

Only six terpenoids and two C_13_-norisoprenoids were detected, and their total concentration was lower than those of the other volatiles (Table 3 and Table 4). However, it has been confirmed that these volatile compounds play significant roles in contributing to the floral aroma of wine due to their low odor threshold (β-ionone for example has an odor threshold of 0.09 µg/L) [29]. Styrene was the dominant volatile in this aroma group. However, no significant concentration differences were observed in these fermented wines. The dried goji berry fermented wine showed higher levels of α-ionene, α-cedrene, α-calacorene, β-ionone, and 2,6-dimethyl-2,6-undecadien-10-ol (0.5 to 7.1 μg/L). As reported, β-ionone can be formed by β-carotene degradation during heat treatment [36]. Obviously, the drying process resulted in the higher content of β-ionone in the dried berry and dried berry fermented wine. The average concentration of β-ionone in the dried berry fermented wine was about 7 μg/L, about two times higher than that in the fresh berry fermented wine. The fresh goji berry fermented wine contained a higher concentration of 2,6,10,10-tetramethyl-1-oxaspiro[4.5]dec-6-ene. 

### 2.3. Effect of Pressing and Glycosidase on Volatile Composition in Goji Berry Wine

Pressing during wine fermentation process can increase the extraction of secondary metabolites present in fruits [14]. However, the high level of these secondary metabolites in juice can produce a green-herbaceous aroma in wine, reducing its overall pleasant flavor [10]. Bound volatiles in fruits cannot contribute to the overall aroma of wine due to their odorless properties. Glycosidase has the capacity of cleaving the sugar moiety from bound volatiles, resulting in the formation of volatile aglycones. These released volatiles are able to exhibit flavor notes, and thus can enhance the overall aroma of wine [9]. 

#### 2.3.1. Effect on Dried Goji Berry Fermented Wine

The pressing treatment resulted in significant differences in the concentration of 27 volatile compounds in the dried goji berry fermented wines (Table 4). For example, the wine fermented from the dried goji berry free-run juice contained higher concentrations of β-cyclocitral, 3-phenyl-4-hydroxyacetophenone, isopentyl acetate, ethyl octanoate, isobutyl acetate, isobutyl hexanoate, isoamyl octanoate, 1-[2-(isobutyryloxy)-1-methylethyl]-2,2-dimethylpropyl 2-methylpropanoate, styrene, and cedrol. Meanwhile, the pressing treatment significantly enhanced the concentrations of decanoic acid, 2-heptanol, 2-ethyl-1-hexanol, 2-undecanone, ethyl caprate, ethyl laurate, hexyl acetate, methyl caprate, diethyl succinate, isoamyl decanoate, and 2,6-dimethyl-2,6-undecadien-10-ol in the dried goji berry fermented wine. Decanoic acid (fatty and rancid odor) [37], ethyl caprate (fruity note) [37], and isopentyl acetate (fruity note) [38] exhibited concentrations higher than their thresholds in the dried goji berry fermented wine with the pressing treatment. The glycosidase treatment did not show a significant effect on the alteration of the aromatic profile in the dried goji berry fermented wine. For example, the increased contents of 1-octen-3-ol, 1-nonanol, and decanal in the dried goji berry fermented wine after the glycosidase treatment were not above their thresholds. The interactive effect from pressing and glycosidase treatments was also limited.

#### 2.3.2. Effect on Fresh Goji Berry Fermented Wine

The pressing treatment showed a more obvious effect on the aromatic composition alteration in the fresh goji berry wine fermentation. For instance, 44 volatile compounds exhibited significant changes on their concentration in the wine after the pressing treatment (Table 4). More importantly, the majority of these volatile compounds were the dominant aromatic compounds in the wine. For example, 2-methyl-1-propanol, ethyl acetate, isopentyl alcohol, and 2,3-butanediol showed their highest contents in the fresh goji berry fermented wine after pressing treatment. 1-Hexanol is considered a significant aroma enhancer, with a concentration of 20-100 mg/L in wine [39,40]. The pressing treatment significantly increased its concentration in the wine, which could contribute its positive odor features in the wine aroma. Glycosidase did not significantly enhance the aromatic levels in the fresh goji berry fermented wine. Six volatiles showed concentration differences in the wines with and without the glycosidase treatment. However, none of them exhibited an OAV value above 1. No significant effect of the interaction between pressing and glycosidase treatments was observed either. 

We speculate that the fresh goji berries, compared to the dried goji berries, possessed more intact cell structures, which limits the extraction of volatile compounds and their precursors in the fresh berries during the vinification process. However, the pressing treatment might destroy the cell structure to release more aromatic compounds and their precursors, increasing their levels in the wine. Additionally, we also speculate that bound volatiles might exist in low levels in goji berries, since the application of glycosidase did not exert a significant effect on the release of more algycones in the goji berry wines. It should be noted that the glycosidase AR2000 used in this study is a non-specific glycosidase that possesses α-l-arabinofuranosidase, α-l-arabinopyranosidase, α-l-rhamnosidase, and α-d-glucosidase activity [41].

### 2.4. Principal Component Analysis (PCA) 

**Figure 1:**
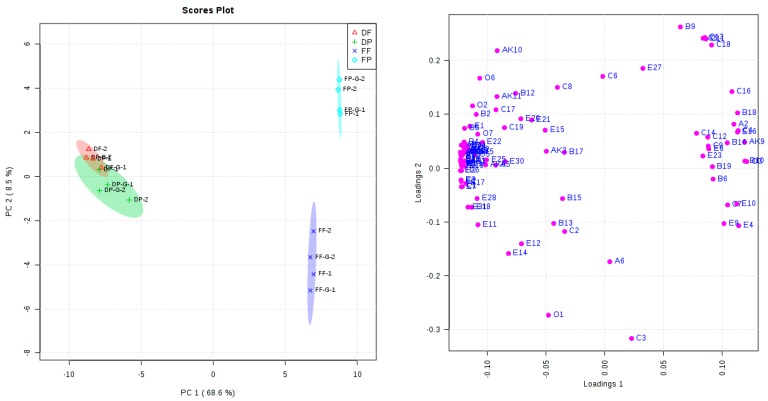
Principal component analysis (PCA) of goji berry fermented wine made of fresh and dried goji berry under pressing and glycosidase treatment. Left—Scores plot; Right—Loading plot; Volatiles with abbreviation of A1-O8 in loading plot are named in Table 3. “1”, “2” in scores plot represent two replicates for each goji berry fruit wine fermentation treatment.

The differentiation of these wines regarding their aromatic profile was observed in the principal component analysis (PCA) with the first two principal components (PC1 and PC2, Figure 1). PC1 represented 68.6% of the total variance, whereas 8.5% of the variance was explained by PC2. PC1 significantly differentiated between the wines made from dried and fresh goji berries due to the negative relation with 20 esters, nine benzenes, eight aldehydes/ketones, six other volatiles, three acids, and two alcohols (volatiles with a loading value below −0.1). Meanwhile, this differentiation was also positively related with four esters, three benzenes, three alcohols, two acids, one aldehyde/ketone, and one other volatile (volatiles with a loading value above 0.1). In addition, a distinction between the fresh goji berry fermented wine made of the free-run and the pressed juice was observed in PC2 according to the relation with isopentyl alcohol (loading value, −0.3166), styrene (−0.2733), benzyl alcohol (0.26244), 1-octanol (0.24277), (*E*)-5-decen-1-ol (0.22878), 1-hexanol (0.2413), and β-cyclocitral (0.21834). The PCA analysis indicated that the goji raw material (fresh vs dried) exerted a primary role in differentiating the aromatic profile of these wines, whereas pressing treatment affected the volatiles profiles of these wines.

### 2.5. Aroma Profile of Goji Berry Fermented Wine

The aroma profile of the goji berry fermented wine was established by calculating the total OAV of the odorants, which was used to better understand its aromatic feature. The aromatic odorants were grouped into fruity, floral, herbaceous (or vegetal), caramel, fatty, and chemical aroma series. In the goji berry fermented wine, 17 volatile compounds showed concentrations higher than their odor threshold. These volatiles included six esters, five alcohols, three acids, one C_13_-norisoprenoid, and one volatile phenol (Table 5). Particularly, β-ionone showed the highest OAV value in the wine among these odorants, indicating that its violet note might be significantly incorporated into the goji berry fermented wine [32]. Isopentyl acetate reached the highest OAV value in the dried goji berry fermented wine, indicating that sweet and fruity notes could be enhanced in the wine [38]. The fresh goji berry fermented wine showed a strong horsy, leather, medicinal, smoky, barnyard, animal, and sweaty odor due to the high concentration of 4-ethylphenol [42,43,44]. In addition, there were 11 volatiles in the wine with its OAV value between 0.1 and 1. These indicated that these volatiles might not directly contribute to the overall aroma of wine but just improve the wine aroma complexity [29].

**Figure 2:**
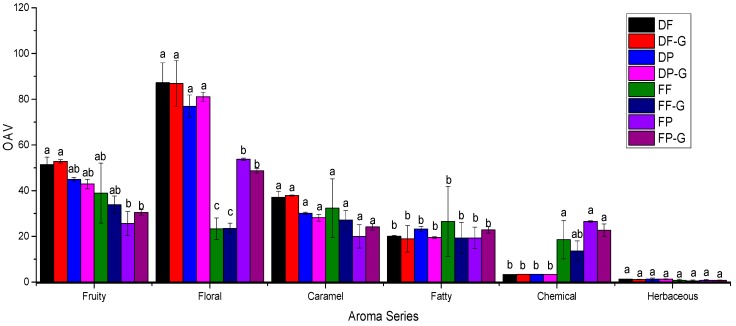
Total OAV value in each aroma group in goji berry fermented wine. The OAV value is calculated by summing up the OAV value of volatile in each aroma group. Different letters indicate significant difference of these wines in each aroma group at *p* ≤ 0.05 of aroma series of goji wines.

Regarding the specific aroma group (Figure 2), the goji berry fermented wine expressed floral aroma features, followed by fruity and caramel notes. These aroma attributes mainly resulted from the predominance of β-ionone and esters in the wine. 4-Ethylphenol and 4-vinylguaiacol featured chemical notes [44]. The fresh goji berry fermented wine, especially the wine obtained after the pressing treatment, exhibited a much stronger chemical odor due to the higher concentration of 4-ethylphenol and 4-vinylguaiacol. Moreover, the dried goji berry fermented wines showed significantly stronger floral odors, and high levels of fruity and caramel aromas. It was also worth noting that these features were much more obvious in the wines fermented from the free-run juice. These indicated that the better aromatic quality of goji berry wine was made from the free-run juice of the dried goji berry. These goji berry fermented wines exhibited little herbaceous flavor notes, and no significant differences were found among these wines.

## 3. Materials and Methods 

### 3.1. Samples and Reagents

Fully ripe goji berries (*Lycium barbarum* L.) were harvested in the Ningxia growing region (Yinchuan City, 106.27°E, 28.47°N) by Senmiao Corporation (Yinchuan, China) in the middle of July of 2015. The fresh goji berries were processed by Senmiao Corporation into dry goji berries through a hot-air drying process as well. Both fresh and the dry goji berries were used for the goji berry wine-making. A commercial Saccharomyces cerevisiae strain Red Fruit^®^, pectinase, and potassium metabisulfite were purchased from Enartis (San Martino, Trecate, Italy). Glycosidase AR 2000 was a product from DSM Food Specialties (Delft, The Netherlands). Deionized water was purified by a Milli-Q purification system (Millipore, Bedford, MA, USA). Sodium hydroxide, diammonium phosphate, sodium chloride, ammonium sulfate, sodium sulfate, tartaric acid, ethanol, and sucrose were purchased from Beijing Chemical Works (Beijing, China). The standards of volatiles were purchased from Sigma-Aldrich (St. Louis, MO, USA).

### 3.2. Wine-Making Process

#### 3.2.1. Wine Made of Dried Goji Berry

Dried goji berries (40 kg, about 75% water loss) were mixed with water (120 L) in a 200 L stainless steel fermenter. Before the physicochemical parameter adjustment, the dried goji berry must had a sugar content of 180.5 glucose equivalent g/L, titratable acidity of 8.6 g/L, and a pH of 4.76. Afterwards, 4 g/hL pectinase and 45 mg/L potassium metabisulfite were added to the fermenter. The resultant mixture was maintained in the fermenter at 10 °C for 24 h, followed by mixing with diammonium phosphate (0.21 g/L). After adjusting the sugar content to 230 g/L using sucrose and pH to 3.8 using tartaric acid, the activated Red Fruit^®^ *S. cerevisiae* (20 g/hL) was added to initiate fermentation. The fermentation was maintained at 20 °C, and monitored by measuring the relative density of the must. When the relative density reached around 1.030, the goji berry juice was separated from the pomace as the free-run juice and transferred to another fermenter to continue the fermentation. The pomace was pressed three times under a 0.8 Mpa for 5 min each time on a pneumatic presser (KSC125X400, Tungming Pneumatic Co., Ltd., Dongguan, China) to yield the pressed juice. Afterwards, the pressed juice was separated from the pomace and maintained in the fermenter for the fermentation. The fermentation of the free-run juice and the pressed juice was considered terminated when the density remained the same in three consecutive days to produce the dried goji berry free-run juice fermented wine (**DF**) and the dried goji berry pressed juice fermented wine (**DP**). These wines were then stored for 1 month at 2 °C in the fermenter filled with CO_2_ for better clarity, and then filtered through 0.45 µm filters. Afterwards, the DF and the DP wine were treated with 4 g/hL glycosidase AR 2000 at 15 °C in the darkness for one week to result in the glycosidase treated dried goji berry free-run juice fermented wine (**DF-G**) and the glycosidase treated dried goji berry pressed juice fermented wine (**DP-G**) according to our preliminary study. Each fermentation was conducted in duplicate, and a total of 8 goji berry wine samples were obtained, including DF (DF-1 and DF-2 as duplicates of DF), DP (DP-1 and DP-2), DF-G (DF-G-1 and DF-G-2), and DP-G (DP-G-1 and DP-G-2).

#### 3.2.2. Wine Made of Fresh Goji Berry 

The fresh goji berry fermentation was conducted using a similar approach as the dried goji berry wine fermentation. Before the adjustments, the fresh goji berry fruit juice had a sugar content of 193.5 g glucose equivalent/L, titratable acidity of 8.4 g/L, and a pH of 4.83. Briefly, the fresh berries (160 kg) were crushed to yield the fresh goji berry must. The must was transferred into a 200 L stainless steel fermenter. Water (10 L) was added to the must to help mix it with the enzyme for a better fermentation performance. Afterwards, 4 g/hL pectinase and 45 mg/L potassium metabisulfite was immediately added. The resultant mixture was maintained in the fermenter at 10 °C for 24 h. Afterwards, 230 g/L sucrose was adjusted and the pH was adjusted to 3.8 using tartaric acid. The fermentation was initiated by adding activated Red Fruit^®^ *S. cerevisiae* (20 g/hL) to the must and maintained at 20 °C. The fresh goji berry free-run juice was collected when the relative density reached around 1.030. The free-run juice was transferred to another fermenter for the fermentation. The fresh goji berry pressed juice was collected using the same pressing procedure applied to the dried goji fruit fermentation, and then fermented in the same fermenter. The fresh goji berry free-run juice fermented wine (**FF**) and the fresh goji berry pressed juice fermented wine (**FP**) were yielded after their density remained the same in three consecutive days of the fermentation. These wines were then stored for 1 month at 2 °C in the fermenter filled with CO_2_ for better clarity, and then filtered through 0.45 µm filters. Afterwards, the FF and the FP wine were treated with 4 g/hL glycosidase AR 2000 at 15 °C in the darkness for one week to yield the glycosidase treated fresh goji berry free-run juice fermented wine (**FF-G**) and the glycosidase treated fresh goji berry pressed juice fermented wine (**FP-G**), respectively. Each fermentation was conducted in duplicate, and a total of 8 goji berry wine samples were obtained, including FF (FF-1 and FF-2 as duplicates of DF), FP (FP-1 and FP-2), FF-G (FF-G-1 and FF-G-2), and FP-G (FP-G-1 and FP-G-2).

The ethanol content of the samples were analyzed by a FOSS WineScan instrument (FOSS, Hillerød, Denmark). The physicochemical parameters of the goji wines, including titratable and volatile acidity, residual sugar content, total and free SO_2_ and pH were determined according to the National Standard of the People’s Republic of China (GB/15038-2006, 2006). The polysaccharide content of the wines was analyzed according to the phenol-sulfuric acid method [50]. The physicochemical parameters of the wines were listed in Table 1.

### 3.3. Volatile Compound Analyses

A headspace solid-phase micro-extraction was used to extract volatile compounds from the wine according to a published method [51]. Briefly, the wine sample (5.0 mL) was mixed with NaCl (1 g) and 1.0018 g/L 4-methyl-2-pentanol (the internal standard, 10 µL) in a 15 mL vial containing a stirring bar. The vial was then capped with a PTFE-silicon septum and heated at 40 °C for 30 min under agitation. Afterwards, a SPME fiber (50/30 µm DVB/Carboxen/PDMS, Supelco, Bellefonte, PA, USA) was placed into the head-space of the mixture in the vial to extract the volatile compounds for 30 min under the same agitation and temperature. Finally, the fiber was removed from the head-space of the mixture and immediately inserted into the GC injector for volatile desorption for 8 min. An Agilent 6890 GC interfaced with an Agilent 5975B MS (Agilent Technologies, Santa Clara, CA, USA) was used to analyze the volatile composition in the wine. The volatile compounds were separated on a HP-INNOWAX capillary column (60 m × 0.25 mm id, 0.25 µm film thickness, J&W Scientific, Folsom, CA, USA) under a carrier gas (helium) flow rate at 1 mL/min with a splitless GC inlet mode. The oven temperature program was as follows: 50 °C held for 1 min and then rose to 220 °C at 3 °C /min and held at 220 °C for 5 min. A voltage of 70 eV was set in the electron impact mode and the mass scan was conducted from *m/z* 20–450 using a selective ion mode. The analysis for each wine sample was conducted in duplicate.

A C_7_–C_24_ *n*-alkane series (Supelco) and the same chromatographic procedure was used to calculate the retention indices. The identification of volatile compounds with the available standard was performed by matching their retention time and mass spectrum with their corresponding standard. For the volatile compounds without the available standard, these volatiles were tentatively identified according to their mass spectrum and retention indices with the standard NIST 11 library and NIST Standard Reference Database.

The reference standards were dissolved in a synthetic matrix solution containing 9 g/L tartaric acid, 12% (*v*/*v*) ethanol, and 10 g/L glucose. The matrix was adjusted to pH 3.8 using 5 mol/L NaOH, and then 10 μL internal standard solution (4-methyl-2-pentanol, 1.0018 g/L) was added. Regarding the volatile composition, the stock solution was composed of 307,000 μg/L of ethyl acetate, 1140 μg/L of isobutyl acetate, 5540 μg/L of ethyl butanoate, 164,000 μg/L of 2-methyl-1-propanol, 2,022,000 μg/L of isopentyl acetate, 9590 μg/L of 1-butanol, 5,648,000 μg/L of isopentyl alcohol, 832,000 μg/L of ethyl hexanoate, 4088 μg/L of styrene, 4830 μg/L of hexyl acetate, 1018 μg/L of ethyl 3-hexenoate, 312 μg/L of 2-heptanol, 464 μg/L of ethyl enanthate, 368 μg/L of 6-methyl-5-heptene-2-one, 204 mg/L of ethyl lactate, 2,021,000 μg/L of 1-hexanol, 8350 μg/L of 3-hexen-1-ol, 1560 μg/L of caprylic acid methyl ester, 910 μg/L of nonanal, 816,000 μg/L of ethyl caprylate, 1650 μg/L of 1-octen-3-ol, 1049 μg/L of acetic acid, 352 μg/L of sulcatol, 5000 μg/L of isopentyl hexanoate, 5376 μg/L of furfural, 900 μg/L of 2-ethyl-1-hexanol, 2530 μg/L of decanal, 770 μg/L of ethyl nonanoate, 2270 μg/L of ethyl 2-hydroxy-4-methylpentanoate, 1650 μg/L of 1-octanol, 1273 mg/L of 2,3-Butanediol, [S-(R*,R*)]-, 668 μg/L of *trans*-2-octenol, 2030 μg/L of butanoic acid, 81.6 μg/L of ethyl caprate, 324 μg/L of isoamyl caprylate, 79.2 μg/L of diethyl succinate, 2240 μg/L of methionol, 484 μg/L of naphthalene, 4352 μg/L of ethyl salicylate, 1140 μg/L of phenethyl acetate, 10.66 μg/L of ethyl laurate, 119 μg/L of dihydropseudoionone, 1.172 μg/L of bnenzyl alcohol, 81.2 μg/L of phenylethyl alcohol, 18.92 μg/L of octanoic acid, 1600 μg/L of 4-ethylphenol, and 18.36 μg/L of decanoic acid. The resultant solution was diluted into fourteen successive levels and extracted using the same headspace solid-phase micro-extraction method in the wine sample to generate standard curve (peak area ratio of volatile standard over internal standard versus concentration of volatile standard). The quantitation of the volatile compound was based on their corresponding standard. For the volatile without the available standard, the quantitation was conducted using the volatile standard that possessed the similar chemical structure or similar C atom numbers (Table 2).

### 3.4. Odor Activity Value and Aroma Series

Odor activity value (OAV) is an indicator to evaluate the contribution of volatile compound to the overall aroma of wine. The OAV value was calculated as the ratio of the concentration of an individual volatile in wine to its perception threshold.

The volatile compounds were divided into six aromatic groups regarding their odor description [28,37,38,46]. These volatile compounds were described to possess: (1) fatty, (2) caramel, (3) fruity, (4) herbaceous (or vegetal), (5) floral, and (6) chemical odors. The total aroma intensity of each aroma group was calculated by summing up the OAVs of each volatile in the group [28].

### 3.5. Statistical Analysis

The difference of mean caused by different goji raw materials (dried goji berry versus fresh goji berry) was assessed using T-test, whereas two-way analysis of variance was performed to investigate the effect of pressing and glycosidase on the volatile concentration difference using SPSS 21.0 (Chicago, IL, USA). Principal component analysis (PCA) was carried out on MetaboAnalyst 3.0 (http://www.metaboanalyst.ca/) using the volatile compounds with the concentration being statistically different among the samples as the variables [52]. Before the PCA analysis, these variables were normalized by autoscaling.

## 4. Conclusions 

In conclusion, goji berry wine was produced using as raw material fresh and dried goji berry fruits and the effect of pressing and glycosidase addition with regard to the aromatic composition in the wine was investigated. Dried goji berry fermented wine contained more odor active volatiles and expressed stronger aromatic features including floral, fruity, and caramel odors. The main aromatic character in goji berry fermented wine included floral, fruity, and caramel odors, whereas a stronger chemical note was found in fresh goji berry fermented wine. Goji berry wine made of free-run juice exhibited a better aromatic features compared to wine fermented from the pressed juice. Glycosidase did not show any beneficial effects on the aromatic profile of goji berry fermented wine.

## Figures and Tables

**Table 1:** Physicochemical parameters of goji berry fermented wines.

Oenological Parameters	DF	DF-G	DP	DP-G	FF	FF-G	FP	FP-G
**Alcohol (%, *v*/*v*)**	13.0 ± 0.0 a	13.00 ± 0.0 a	12.5 ± 0.2 bcd	12.6 ± 0.0 bc	12.4 ± 0.1 cd	12.7 ± 0.0 b	12.4 ± 0.0 d	12.3 ± 0.0 d
**Volatile acidity (g/L)**	1.2 ± 0.0 b	1.2 ± 0.0 bc	1.1 ± 0.0 e	0.8 ± 0.0 g	1.2 ± 0.0 cd	1.0 ± 0.0 f	1.1 ± 0.0 de	2.1 ± 0.0 a
**Titratable acidity ^a^ (g/L)**	12.2 ± 0.1 ab	11.9 ± 0.1 bc	11.8 ± 0.0 c	12.4 ± 0.1 a	7.9 ± 0.0 e	7.7 ± 0.0 e	8.2 ± 0.1 e	7.8 ± 0.0 d
**Residual sugar (g/L)**	9.3 ± 0.4 a	10.0 ± 0.4 a	10.1 ± 0.7 a	9.1 ± 0.0 a	7.8 ± 0.1 b	6.6 ± 0.0 b	8.4 ± 0.1 b	7.6 ± 0.1 b
**Polysaccharide ^b^ (g/L)**	1.6 ± 0.1 b	1.5 ± 0.0 bc	3.0 ± 0.1a	2.9 ±0.1 a	1.4 ± 0.0 cd	1.2 ± 0.0 df	1.3 ± 0.1 d	1.1 ± 0.1 f
**Free SO_2_ (mg/L)**	3.4 ± 0.0 c	1.7 ± 0.1 d	3.4 ± 0.0 c	1.7 ± 0.0 d	13.4 ± 0.0 a	7.3 ± 0.4 b	13.4 ± 0.0 a	6.7 ± 0.0 b
**Total SO_2_ (mg/L)**	100.4 ± 0.1 f	98.8 ± 0.2 g	93.7 ± 0.0 h	132.1 ± 0.0 d	210.5 ± 0.2 b	153.9 ± 0.1 c	237.5 ± 0.1 a	112.0 ± 0.0 e
**pH**	4.0 ± 0.0 c	3.9 ± 0.0 c	4.0 ± 0.0 b	4.0 ±0.0 b	4.4 ± 0.0 a	4.5 ±0.0 a	4.4 ± 0.0 a	4.5 ± 0.0 a

DF and DP indicate dried goji berry fermented wine made of free-run and pressed juice, respectively. DF-G and DP-G represent dried goji berry fermented wine made of free-run and pressed juice after the glycosidase treatment, respectively. FF and FP are fresh goji berry fermented wine made of free-run and pressed juice, respectively. FF-G and FP-G stand for fresh goji berry fermented wine made of free-run and pressed juice after the glycosidase treatment, respectively. ^a^ Expressed as g/L of malic acid. ^b^ Expressed as g/L of glucose. Different letters in the same row means significant differences at *p* ≤ 0.05. Data were expressed as the mean ± standard deviation.

**Table 2:** Volatile compounds identification and their linearity range.

Volatile	CN ^a^	RI	ID ^b^	QI ^c^	Standard	Quantitative Curve	R^2^	Linearity Range (μg/L)
***Volatile acids***	A							
DMECA ^d^	A1	1367.6	B	95	4-Ethylphenol	y = 233.46x + 1.5085	R^2^ = 0.9967	0.78–50.00
Acetic acid	A2	1453.8	A	43	Acetic acid	y = 53.61x − 29.07	R^2^ = 0.9948	32.78–1049.00
Butanoic acid	A3	1630.4	A	60	Butanoic acid	y = 21574x + 7.5319	R^2^ = 0.9999	126.88–1015.00
α-Methylbutyric acid	A4	1671.7	B	74	Butanoic acid	y = 21814x − 0.9852	R^2^ = 0.9994	63.44–1015.00
Octanoic acid	A5	2062.1	A	60	Octanoic acid	y = 7754.7x + 1401.7	R^2^ = 0.9982	2365.00–9460.00
Decanoic acid	A6	2271.8	A	60	Decanoic acid	y = 55699x − 252.24	R^2^ = 0.9609	1147.50–9180.00
***Higher alcohols***	C							
2-Methyl-1-propanol	C1	1104.0	A	43	2-Methyl-1-propanol	y = 17537x + 5926.7	R^2^ = 0.9861	10250.00–82000.00
1-Butanol	C2	1158.1	A	56	1-Butanol	y = 17266x + 36.061	R^2^ = 1	299.69–1198.75
Isopentyl alcohol	C3	1216.0	A	55	Isopentyl alcohol	y = 9481.2x − 6073.4	R^2^ = 0.9985	35300.00–282400.00
Pentyl alcohol	C4	1255.5	B	42	1-Hexanol	y = 1162.6x − 1.2723	R^2^ = 0.9999	4.93–78.95
1-Hexanol	C5	1352.5	A	56	1-Hexanol	y = 1166.2x − 2.2718	R^2^ = 0.9999	39.47–315.78
(*Z*)-3-Hexen-1-ol	C6	1384.7	A	41	(*Z*)-3-Hexen-1-ol	y = 4651.6x − 7.5812	R^2^ = 0.9968	32.62–260.94
2-Heptanol	C7	1320.0	A	45	2-Heptanol	y = 210.83x + 0.0189	R^2^ = 0.9944	2.44–9.75
3-Ethoxy-1-propanol	C8	1376.9	B	31	1-Hexanol	y = 1161.1x − 1.192	R^2^ = 0.9998	9.87–78.95
1-Octen-3-ol	C9	1448.6	A	57	1-Octen-3-ol	y = 118.3x − 0.4557	R^2^ = 0.9993	3.22–25.78
Sulcatol	C10	1461.0	A	95	Sulcatol	y = 514.36x − 60.573	R^2^ = 0.9936	0.69–11.00
2-Ethyl-1-hexanol	C11	1488.8	A	27	2-Ethyl-1-hexanol	y = 89.473x − 0.447	R^2^ = 0.9988	0.44–14.06
[*R*-(R*,R*)]-2,3-Butanediol	C12	1538.9	B	45	[*S*-(R*,R*)]-2,3-Butanediol	y = 82896x − 16783	R^2^ = 0.9993	39781.25–636500.00
1-Octanol	C13	1557.0	A	56	1-Octanol	y = 182.89x + 0.2501	R^2^ = 0.9996	6.45–51.56
[*S*-(R*,R*)]-2,3-Butanediol	C14	1574.9	A	45	[*S*-(R*,R*)]-2,3-Butanediol	y = 82896x − 16783	R^2^ = 0.9993	39781.25–636500.00
*trans*-2-Octenol	C15	1616.0	A	57	*trans*-2-Octenol	y = 258.79x + 0.2617	R^2^ = 0.9977	2.61–20.88
1-Nonanol	C16	1659.1	B	56	Acetic acid	y = 10.169x − 0.0206	R^2^ = 0.9856	0.13–0.51
Methionol	C17	1720.6	A	106	Methionol	y = 34890x − 113.09	R^2^ = 0.9962	280.00–1120.00
*E*-5-Decen-1-ol	C18	1795.2	B	67	1-Dodecanol	y = 300.63x + 0.7633	R^2^ = 0.9962	2.52–20.16
Phenylethyl alcohol	C19	1920.2	A	91	Phenylethyl alcohol	y = 1833.3x − 518.58	R^2^ = 0.9940	2537.50–40600.00
***Aldehydes/Ketones***	AK							
***Aldehydes***								
Nonanal	AK1	1399.2	A	57	Nonanal	y = 97.464x − 2.3267	R^2^ = 0.9929	0.44–28.44
Furfural	AK2	1471.8	A	96	Furfural	y = 1798.6x − 2.8178	R^2^ = 0.9987	5.25–84.00
Decanal	AK3	1504.4	A	43	Decanal	y = 120.85x + 0.8893	R^2^ = 0.9878	0.31–39.53
Safranal	AK4	1656.8	B	107	α-Terpineol	y = 34.957x + 0.2522	R^2^ = 0.9950	0.42–3.38
2-Butyl-2-octenal	AK5	1675.4	B	41	Ethyl caprate	y = 277.88x − 4.4049	R^2^ = 0.9917	4.98–39.84
***Ketones***								
Isobutyl ketone	AK6	1194.0	B	57	6M5H2NE	y = 243.64x − 5.3362	R^2^ = 0.9960	0.04–92.00
TMCHN ^e^	AK7	1325.0	B	82	6M5H3NE	y = 275.14x − 6.922	R^2^ = 0.9947	0.72–11.50
6M5H2NE ^f^	AK8	1344.7	A	43	6M5H4NE	y = 278.06x − 7.1512	R^2^ = 0.9971	0.36–23.00
2-Undecanone	AK9	1603.7	B	58	Dihydropseudoionone	y = 53.532x + 0.0331	R^2^ = 0.9995	0.93–7.44
β-Cyclocitral	AK10	1630.9	B	137	α-Terpineol	y = 179.5x + 0.7247	R^2^ = 0.9993	1.91–7.63
Dihydropseudoionone	AK11	1861.4	A	43	Dihydropseudoionone	y = 50.565x + 0.0693	R^2^ = 0.9981	0.23–14.88
2-Acetylpyrrole	AK12	1981.6	B	94	Hexanal	y = 1093.5x − 17.772	R^2^ = 0.9994	30.74–245.94
TMHAAL ^g^	AK13	2372.3	B	111	4-Ethylphenol	y = 312.57x + 0.2238	R^2^ = 0.9959	0.10–12.50
***Benzenes/Phenols***	B							
***Benzenes***								
Isodurene	B1	1498.2	B	119	*p*-Cymene	y = 54.679x − 0.1459	R^2^ = 0.9956	0.41–3.28
TEMLNE ^h^	B2	1677.5	B	159	Ethyl salicylate	y = 79.909x + 0.0671	R^2^ = 0.9932	0.53–17.00
DPPXL ^i^	B3	1694.1	B	175	Ethyl salicylate	y = 56.72x + 3.6823	R^2^ = 0.9945	8.50–34.00
Naphthalene	B4	1756.0	A	128	Naphthalene	y = 10.996x + 0.5718	R^2^ = 0.9915	0.95–7.56
TMDHPE ^j^	B5	1758.3	B	157	Ethyl salicylate	y = 88.273x − 0.0789	R^2^ = 0.9900	4.98–39.84
M2PB ^k^	B6	1790.2	B	120	Ethyl phenacetate	y = 35.188x + 0.2362	R^2^ = 0.9924	0.84–3.38
Phenethyl acetate	B7	1826.3	A	104	Phenethyl acetate	y = 69.63x + 5.0169	R^2^ = 0.9996	17.81–285.00
β-Methylnaphthalene	B8	1870.0	B	142	Ethyl salicylate	y = 60.837x + 1.9511	R^2^ = 0.9906	2.13–34.00
Benzyl alcohol	B9	1883.4	A	79	Benzyl alcohol	y = 3642.2x + 19.022	R^2^ = 0.9979	91.56–732.50
Ethyl dihydrocinnamate	B10	1895.6	B	104	Ethyl phenacetate	y = 29.931x + 0.4139	R^2^ = 0.9944	0.42–6.75
α-Methylnaphthalene	B11	1906.9	B	142	Ethyl salicylate	y = 79.909x + 0.0671	R^2^ = 0.9932	0.53–17.00
4-Tolylcarbinol	B12	1981.6	B	107	4-Ethylphenol	y = 391.78x − 0.0605	R^2^ = 0.9863	0.20–3.13
Ethyl cinnamate	B13	2145.3	B	131	Ethyl phenacetate	y = 29.468x + 0.4874	R^2^ = 0.9940	0.84–6.75
3P4HP ^l^	B14	2249.8	B	197	Ethyl phenacetate	y = 34.957x + 0.2522	R^2^ = 0.9950	0.42–3.38
DTBPH ^m^	B15	2308.7	B	191	4-Ethylphenol	y = 166.76x + 11.394	R^2^ = 0.9934	25.00–100.00
Coumaran	B16	2403.1	B	120	4-Ethylphenol	y = 159.63x + 29.604	R^2^ = 0.9862	12.50–800.00
***Phenols***								
Ethyl salicylate	B17	1824.6	A	120	Ethyl salicylate	y = 60.837x + 1.9511	R^2^ = 0.9906	2.13–34.00
4-Ethylphenol	B18	2182.8	A	107	4-Ethylphenol	y = 162.82x + 18.57	R^2^ = 0.9863	1.56–800.00
4-Vinylguaiacol	B19	2208.5	B	135	4-Ethylphenol	y = 141.15x + 101.45	R^2^ = 0.9973	200.00–800.00
***Esters*** ***Ethyl esters***	E							
Ethyl Acetate	E1	764.8	A	43	Ethyl Acetate	y = 6076.6x − 2518.1	R^2^ = 0.9978	19187.50–153500.00
Ethyl butanoate	E2	1037.5	A	71	Ethyl butanoate	y = 613.77x + 1.0444	R^2^ = 0.9999	5.41–692.50
Ethyl hexanoate	E3	1244.4	A	88	Ethyl hexanoate	y = 68.562x − 6.8083	R^2^ = 1	40.63–650.00
Ethyl 3-hexenoate	E4	1307.6	A	69	Ethyl 3-hexenoate	y = 80.813x − 0.0169	R^2^ = 0.9976	0.62–9.94
Ethyl enanthate	E5	1337.6	A	88	Ethyl enanthate	y = 35.894x + 0.1203	R^2^ = 0.9997	0.91–3.63
Ethyl lactate	E6	1346.8	A	45	Ethyl lactate	y = 28150x − 92.569	R^2^ = 0.9960	199.22–6375.00
Ethyl octanoate	E7	1438.7	A	88	Ethyl octanoate	y = 42.269x + 91.381	R^2^ = 0.9998	318.75–1275.00
Ethyl mesitylacetate	E8	1511.8	B	133	Ethyl phenacetate	y = 38.305x + 0.1918	R^2^ = 0.9779	0.42–1.69
Ethyl nonanoate	E9	1539.5	A	88	Ethyl nonanoate	y = 42.505x + 0.9106	R^2^ = 0.9940	1.50–12.03
E2H4MP ^n^	E10	1546.9	A	69	E2H4MP	y = 394.42x + 1.2915	R^2^ = 0.9999	8.87–35.47
Ethyl caprate	E11	1642.8	A	88	Ethyl caprate	y = 78.134x + 132.78	R^2^ = 0.9911	159.38–1275.00
Ethyl benzoate	E12	1677.4	B	105	Ethyl phenacetate	y = 35.188x + 0.2362	R^2^ = 0.9924	0.84–3.38
Ethyl 9-decenoate	E13	1694.3	B	55	Ethyl laurate	y = 278.11x + 0.9429	R^2^ = 0.9891	0.65–41.64
Ethyl laurate	E14	1847.5	A	88	Ethyl laurate	y = 97.913x + 53.207	R^2^ = 0.9985	83.28–333.13
Ethyl myristate	E15	2053.2	B	88	Ethyl laurate	y = 255.61x + 3.5176	R^2^ = 0.9965	10.41–41.64
Ethyl hexadecanoate	E16	2256.1	B	88	Ethyl laurate	y = 140.86x + 16.882	R^2^ = 0.9696	10.41–166.56
***Acetates***								
Isobutyl acetate	E17	1009.6	A	43	Isobutyl acetate	y = 417.2x − 134.5	R^2^ = 0.9996	17.81–570.00
Isopentyl acetate	E18	1135.9	A	43	Isopentyl acetate	y = 186.69x − 260.63	R^2^ = 0.9983	631.88–10110.00
Hexyl acetate	E19	1298.8	A	43	Hexyl acetate	y = 44.969x − 0.3454	R^2^ = 0.9999	0.29–75.47
Heptyl acetate	E20	1377.4	B	43	Hexyl acetate	y = 46.025x − 0.4832	R^2^ = 0.9999	2.36–18.87
2-Ethyl-1-hexanol acetate	E21	1387.2	B	43	2-Heptanol	y = 224.82x − 0.2812	R^2^ = 0.9985	1.22–19.50
Octyl acetate	E22	1477	B	43	2-Heptanol	y = 224.82x − 0.2812	R^2^ = 0.9985	1.22–19.50
Trimethylene acetate	E23	1740.3	B	43	Diethyl succinate	y = 749.61x + 12.249	R^2^ = 0.9987	38.67–309.38
***Other esters***								
Isobutyl hexanoate	E24	1357.7	B	99	Styrene	y = 54.92x − 0.4452	R^2^ = 0.9928	0.25–3.99
Methyl octanoate	E25	1393.8	A	74	Methyl octanoate	y = 23.885x + 0.7001	R^2^ = 0.9991	0.76–48.75
Isopentyl hexanoate	E26	1462.3	A	70	Isopentyl hexanoate	y = 58.981x + 0.1264	R^2^ = 0.9945	0.61–9.77
Isobutyl octanoate	E27	1554.6	B	57	Isoamyl caprylate	y = 33.7x + 0.8987	R^2^ = 0.9878	0.63–10.13
Methyl caprate	E28	1599.0	B	74	Ethyl caprate	y = 277.88x − 4.4049	R^2^ = 0.9917	4.98–39.84
Isoamyl octanoate	E29	1662.7	A	70	Isoamyl octanoate	y = 25.546x + 1.5448	R^2^ = 0.9815	0.63–20.25
Diethyl succinate	E30	1680.1	A	101	Diethyl succinate	y = 709.47x + 17.935	R^2^ = 0.9991	38.67–618.75
Isoamyl decanoate	E31	1866.8	B	70	Isoamyl caprylate	y = 32.527x + 1.1504	R^2^ = 0.9920	1.27–10.13
IMDMMP ^o^	E32	1883.2	B	71	Isoamyl caprylate	y = 25.382x + 1.5173	R^2^ = 0.9829	0.63–20.25
NA3MBE ^p^	E33	1905.7	B	141	Isoamyl caprylate	y = 32.527x + 1.1504	R^2^ = 0.9920	1.27–10.13
***Others***	O							
Styrene	O1	1269.8	A	104	Styrene	y = 37.916x + 1.5324	R^2^ = 0.9989	31.94–127.75
α-Ionene	O2	1489.7	B	159	Ethyl salicylate	y = 60.837x + 1.9511	R^2^ = 0.9906	2.13–34.00
ODETM ^q^	O3	1549.5	B	138	Ethyl salicylate	y = 78.368x + 0.3169	R^2^ = 0.9952	1.06–17.00
α-Cedrene	O4	1625.9	B	119	E-Nerolidol	y = 12.967x + 0.0836	R^2^ = 0.9864	0.03–2.13
α-Calacorene	O5	1931.9	B	157	Ethyl salicylate	y = 76.261x + 0.6681	R^2^ = 0.9980	2.13–17.00
β-Ionone	O6	1951.7	B	177	Ethyl salicylate	y = 78.368x + 0.3169	R^2^ = 0.9952	1.06–17.00
DMUDL ^r^	O7	1955.5	B	109	E-Nerolidol	y = 16.415x + 0.0339	R^2^ = 0.9952	0.07–1.06
Cedrol	O8	2137.5	B	95	E-Nerolidol	y = 15.972x + 0.0556	R^2^ = 0.9981	0.13–1.06

^a^ Code name. ^b^ Identification of the compounds: “A” means compound identified by mass spectrum and RI according to the standard, whereas “B” represents compound tentatively identified by mass spectrum with database and RI according to literatures. ^c^ Quantitation ion. ^d^ 1,2-Dimethyl-cyclopentene-2-enecarboxylic acid. ^e^ 2,2,6-Trimethylcyclohexanone. ^f^ 6-Methyl-5-heptene-2-one. ^g^ (2,6,6-Trimethyl-2-hydroxycyclohexylidene)acetic acid lactone. ^h^ 1,1,4,6-Tetramethylindane. ^i^ 2,5-Diisopropyl-*p*-xylene. ^j^ 2,5,8-Trimethyl-1,2-dihydronaphthalene. ^k^ Methyl 2-(pentyloxy)benzoate. ^l^ 3-Phenyl-4-hydroxyacetophenone. ^m^ 2,4-Di-*tert*-butylphenol. ^n^ Ethyl 2-hydroxy-4-methylpentanoate. ^o^ 1-[2-(Isobutyryloxy)-1-methylethyl]-2,2-dimethylpropyl 2-methylpropanoate. ^p^ Nonanoic acid, 3-methylbutyl-2 ester. ^q^ 1-Oxaspiro[4.5]dec-6-ene, 2,6,10,10-tetramethyl-. ^r^ 2,6-Dimethyl-2,6-undecadien-10-ol.

**Table 3:** Concentration of volatile compounds in goji berry fermented wine.

Volatile (µg/L)	DF	DF-G	DP	DP-G	FF	FF-G	FP	FP-G
***Volatile acids***								
DMECAd	40.8 ± 1.5 a *	41.2 ± 1.0 a	39.8 ± 0.7 a	41.3 ± 1.4 a	2.5 ± 0.0 b	2.5 ± 0.1 b	2.6 ± 0.2 b	2.4 ± 0.1 b
Acetic acid	172.6 ± 53.0 bc	199.8 ± 96.6 bc	135.5 ± 81.7 c	177.3 ± 32.0 bc	469.4 ± 122.5 abc	417.6 ± 86.1 abc	639.3 ± 121.1 a	525.3 ± 101.0 ab
Butanoic acid	1506.8 ± 135.7 a	1489.4 ± 359.1 a	1488.7 ± 213.3 a	1503.4 ± 69.9 a	414.3 ± 99.4 b	412.4 ± 13.0 b	418.2 ± 71.9 b	373.7 ± 5.8 b
α-Methylbutyric acid	1532.2 ± 42.6 a	1450.2 ± 188.3 a	1544.0 ± 210.31 a	1519.5 ± 14.8 a	531.2 ± 104.0 b	488.5 ± 6.4 b	539.4 ± 9.3 b	473.4 ± 5.3 b
Octanoic acid	1018.7 ± 49.8 a	819.8 ± 297.4 a	1045.5 ± 122.6 a	914.2 ± 10.2 a	740.8 ± 318.8 a	598.5 ± 172.9 a	610.5 ± 39.9 a	563.6 ± 38.0 a
Decanoic acid	2712.4 ± 146.2 a	2186.8 ± 995.4 a	6053.6 ± 1901.4 a	3325.7 ± 203.0 a	5047.2 ± 1410.4 a	4215.8 ± 1303.4 a	2570.7 ± 28.2 a	2752.2 ± 385.4 a
*Total*	*6983.5 ± 340.5 ab*	*6187.3 ± 1936.0 ab*	*10306.8 ± 1274.1 a*	*7481.4 ± 98.0 ab*	*7205.3 ± 2055.1 ab*	*6135.4 ± 1568.9 ab*	*4780.6 ± 195.3 b*	*4690.6 ± 235.5 b*
***Higher alcohols***								
2-Methyl-1-propanol	35548.7 ± 1625.3 a	35130.6 ± 589.8 a	36260.1 ± 1605.77 a	35318.8 ± 1043.7 a	23463.5 ± 907.0 b	23601.0 ± 687.37 b	20073.4 ± 98.2 b	20012.0 ± 418.5 b
1-Butanol	967.2 ± 87.1 a	955.0 ± 104.7 a	948.5 ± 42.7 a	953.4 ± 10.5 a	948.0 ± 33.48 a	978.0 ± 61.4 a	929.3 ± 103.8 a	866.2 ± 16.9 a
Isopentyl alcohol	187579.3 ± 2742.2 b	184874.0 ± 6446.52 b	188753.9 ± 5009.5 b	183698.5 ± 816.3 b	217389.5 ± 7670.1 a	214993.6 ± 267.8 a	173630.3 ± 1822.6 b	173019.9 ± 48.9 b
Pentyl alcohol	17.0 ± 3.19 c	21.4 ± 3.1 bc	17.9 ± 3.3 c	15.7 ± 0.3 c	28.1 ± 1.1 ab	27.5 ± 3.0 ab	32.1 ± 0.2 a	34.3 ± 1.8 a
1-Hexanol	91.5 ± 3.0 b	99.3 ± 5.3 b	92.8 ± 6.5 b	90.5 ± 5.1 b	94.5 ± 2.7 b	101.5 ± 1.7 b	160.7 ± 1.0 a	169.1 ± 2.7 a
(*Z*)-3-Hexen-1-ol	123.8 ± 21.6 a	132.7 ± 5.2 a	134.0 ± 4.58 a	115.6 ± 8.4 a	100.3 ± 0.4 a	122.4 ± 21.7 a	129.8 ± 32.3 a	144.4 ± 29.2 a
2-Heptanol	5.6 ± 0.1 c	5.7 ± 0.3 c	6.3 ± 0.2 bc	6.3 ± 0.1 bc	7.8 ± 0.3 a	7.3 ± 0.1 ab	6.7 ± 0.1 abc	7.8 ± 0.6 a
3-Ethoxy-1-propanol	39.9 ± 1.6 a	35.1 ± 15.8 a	33.7 ± 9.1 a	32.6 ± 1.1 a	32.5 ± 3.1 a	26.4 ± 6.1 a	34.9 ± 2.9 a	32.4 ± 3.8 a
1-Octen-3-ol	6.8 ± 0.6 a	7.6 ± 0.1 a	6.9 ± 0.3 a	8.0 ± 0.56 a	9.4 ± 3.8 a	13.6 ± 0.1 a	13.9 ± 0.5 a	13.2 ± 8.1 a
Sulcatol	TR **	TR	TR	TR	TR	TR	TR	TR
2-Ethyl-1-hexanol	12.2 ± 0.2 f	13.0 ± 0.4 ef	16.2 ± 1.2 cd	15.4 ± 0.3 de	19.4 ± 1.4 b	18.4 ± 0.2 bc	75.5 ± 0.5 a	76.0 ± 0.2 a
[*R*-(R*,R*)]-2,3-Butanediol	375898.6 ± 82505.1 a	363649.8 ± 241292.74 a	348050.8 ± 196950.2 a	279674.0 ± 61987.2 a	1766887.6 ± 1645173.9 a	979257.2 ± 660565.6 a	1313477.4 ± 699634.8 a	1735789.3 ± 233651.6 a
1-Octanol	10.9 ± 2.9 b	11.1 ± 2.0 b	9.3 ± 2.1 b	10.2 ± 1.2 b	12.3 ± 0.8 b	11.8 ± 0.5 b	31.9 ± 0.7 a	33.1 ± 0.5 a
[*S*-(R*,R*)]-2,3-Butanediol	117488.4 ± 16482.5 a	123910.7 ± 67104.1 a	124577.5 ± 48004.6 a	86202.4 ± 13306.7 a	305149.2 ± 252625.20 a	190949.9 ± 107619.4 a	205124.8 ± 82211.3 a	338514.7 ± 20924.2 a
trans-2-Octenol	9.5 ± 0.7 b	10.3 ± 0.4 b	9.5 ± 0.3 b	10.0 ± 1.21 b	5.3 ± 0.4 a	5.0 ± 0.1 a	4.6 ± 0.0 a	5.9 ± 0.5 a
1-Nonanol	0.1 ± 0.0 c	0.1 ± 0.0 c	0.1 ± 0.0 c	0.1 ± 0.0 c	0.2 ± 0.0 b	0.2 ± 0.0 bc	0.3 ± 0.0 a	0.3 ± 0.0 a
Methionol	1316.9 ± 37.0 a	1162.2 ± 63.3 a	1276.4 ± 540.2 a	1280.3 ± 143.6 a	775.5 ± 483.1 a	544.1 ± 340.8 a	816.2 ± 254.5 a	770.5 ± 168.4 a
*E*-5-Decen-1-ol	ND ***	ND	ND	ND	8.9 ± 1.7 b	7.7± 0.8 b	61.7 ± 1.2 a	58.8 ± 1.1 a
Phenylethyl Alcohol	20978.7 ± 1150.9 a	20111.8 ± 5434.5 a	19807.4 ± 7958.7 a	20117.5 ± 489.0 a	15526.1 ± 8671.7 a	11607.3 ± 4687.1 a	14267.9 ± 1231.0 a	13133.9 ± 2106.6 a
*Total*	*740054.1 ± 98959.9 a*	*730092.8 ± 320914.5 a*	*719960.1 ± 246869.2 a*	*607507.9 ± 77776.8 a*	*2330406.2 ± 1915574.3 a*	*1422220.4 ± 773718.3 a*	*1728818.3 ± 781475.9 a*	*2282631.4 ± 257372.4 a*
***Aldehyde/Ketones***								
Nonanal	14.2 ± 0.3 a	15.7 ± 1.2 a	10.7 ± 0.7 a	12.8 ± 0.0 a	5.8 ± 0.3 a	5.1 ± 0.3 a	4.5 ± 0.3 a	4.5 ± 0.9 a
Furfural	492.2 ± 0.8 a	484.6 ± 32.0 a	436.9 ± 38.4 a	465.3 ± 4.0 a	11.7 ± 2.9 b	11.7 ± 3.3 b	12.8 ± 1.6 b	11.8 ± 0.3 b
Decanal	3.7 ± 0.7 a	4.9 ± 1.0 a	2.5 ± 0.0 a	4.5 ± 0.6 a	4.0 ± 0.90 a	2.4 ± 0.3 a	2.4 ± 0.6 a	3.7 ± 1.2 a
Safranal	2.3 ± 0.0 a	2.4 ± 0.2 a	2.2 ± 0.1 a	2.2 ± 0.1 a	0.5 ± 0.0 b	0.5 ± 0.0 b	0.6 ± 0.0 b	0.6 ± 0.0 b
2-Butyl-2-octenal	16.7 ± 1.3 a	17.1 ± 3.1 a	17.4 ± 3.4 a	18.1 ± 0.4 a	11.9 ± 2.4 a	10.6 ± 1.4 a	11.0 ± 1.1 a	10.9 ± 1.3 a
Isobutyl ketone	22.0 ± 5.0 ab	24.0 ± 8.0 ab	7.58 ± 5.93 ab	33.1 ± 13.2 a	TR	2.48 ± 0.37 b	TR	0.09 ± 0.53 b
TMCHN	TR	TR	TR	TR	TR	TR	TR	TR
6M5H2NE	11.0 ± 1.15 a	11.9 ± 4.1 a	8.2 ± 1.1 a	8.0 ± 0.7 a	TR	TR	TR	TR
2-Undecanone	1.9 ± 0.0 b	2.1 ± 0.1 b	2.0 ± 0.1 a	2.0 ± 0.07 a	3.5 ± 0.5 c	3.2 ± 0.1 c	3.6 ± 0.0 d	3.9 ± 0.2 cd
β-Cyclocitral	6.5 ± 0.1 a	6.6 ± 0.4 a	5.6 ± 0.0 b	5.6 ± 0.1 b	3.0 ± 0.2 c	3.4 ± 0.3 c	5.3 ± 0.1 b	4.9 ± 0.1 b
Dihydropseudoionone	4.4 ± 0.4 ab	4.5 ± 0.1 ab	5.5 ± 2.3 a	3.7 ± 0.1 abc	1.3 ± 0.3 cd	0.9 ± 0.1 c	2.7 ± 0.0 abc	2.8 ± 0.1 abc
2-Acetylpyrrole	121.6 ± 8.1 a	116.16 ± 43.2 a	114.3 ± 53.6 a	111.7 ± 0.5 a	ND	ND	ND	ND
TMHAAL	6.8 ± 0.34 a	6.5 ± 1.6 a	7.1 ± 1.6 a	6.0 ± 0.45 a	0.9 ± 0.1 b	1.6 ± 0.5 b	1.4 ± 0.1 b	1.1 ± 0.1 b
*Total*	*702.1 ± 13.8 a*	*696.3 ± 70.2 a*	*618.8 ± 100.2 b*	*671.7 ± 8.3 ab*	*37.4 ± 7.0 c*	*37.4 ± 6.0 c*	*42.3 ± 0.0 c*	*43.4 ± 1.9 c*
***Benzene***								
Isodurene	4.3 ± 0.1 a	4.1 ± 0.2 a	4.6 ± 0.2 a	4.6 ± 0.4 a	1.3 ± 0.0 b	1.3 ± 0.1 b	1.0 ± 0.1 b	0.9 ± 0.0 b
TEMLNE	7.2 ± 0.1 a	5.8 ± 1.4 abc	5.8 ± 2.1 abc	6.9 ± 0.7 ab	2.8 ± 0.6 c	2.2 ± 0.2 c	3.8 ± 0.1 abc	3.1 ± 0.7 bc
DPPXL	31.1 ± 0.9 a	29.2 ± 1.3 ab	24.3 ± 7.7 abc	28.4 ± 2.0 ab	18.4 ± 1.5 bc	18.2 ± 0.1 bc	16.7 ± 0.6 c	16.5 ± 0.6 c
Naphthalene	4.5 ± 0.2 a	5.3 ± 0.4 a	4.5 ± 0.3 a	4.7 ± 0.1 a	2.2 ± 0.3 b	2.0 ± 0.1 b	2.3 ± 0.0 b	2.3 ± 0.0 b
TMDHPE	6.2 ± 0.2 a	5.9 ± 0.1 a	5.3 ± 1.1 a	5.8 ± 0.5 a	1.9 ± 0.2 b	1.6 ± 0.0 b	2.4 ± 0.2 b	2.4 ± 0.3 b
M2PB	1.5 ± 0.1 ab	1.6 ± 0.0 ab	1.5 ± 0.1 ab	1.3 ± 0.0 b	1.9 ± 0.3 a	1.7 ± 0.0 ab	1.7 ± 0.0 ab	1.7 ± 0.1 ab
Phenethyl acetate	144.7 ± 10.9 a	141.0 ± 17.4 a	114.9 ± 15.3 a	122.2 ± 1.0 a	43.6 ± 7.4 b	37.5 ± 2.1 b	28.1 ± 0.7 b	26.4 ± 0.2 b
β-Methylnaphthalene	17.2 ± 0.4 a	16.0 ± 1.6 a	16.0 ± 1.8 a	17.0 ± 0.44 a	5.6 ± 0.7 b	5.4 ± 0.1 b	4.7 ± 0.1 b	4.7 ± 0.1 b
Benzyl alcohol	308.3 ± 2.0 a	297.9 ± 60.4 a	305.6 ± 111.7 a	314.6 ± 7.7 a	314.5 ± 140.8 a	289.1 ± 75.4 a	527.1 ± 23.0 a	514.1 ± 80.8 a
Ethyl dihydrocinnamate	1.2 ± 0.0 b	1.3 ± 0.1 b	1.2 ± 0.0 b	1.3 ± 0.0 b	3.2 ± 0.6 a	2.7 ± 0.2 a	3.2 ± 0.1 a	3.1 ± 0.1 a
α-Methylnaphthalene	11.4 ± 0.2 a	10.8 ± 0.7 a	11.1 ± 1.1 a	11.5 ± 0.3 a	2.7 ± 0.5 b	2.1 ± 0.1 b	2.0 ± 0.1 b	1.9 ± 0.1 b
4-Tolylcarbinol	1.4 ± 0.1 a	1.3 ± 0.3 a	1.4 ± 0.1 a	1.4 ± 0.1 a	0.9 ± 0.9	0.5 ± 0.1	1.0 ± 0.06	1.0 ± 0.0
Ethyl cinnamate	1.9 ± 0.2 a	2.5 ± 0.5 a	2.4 ± 0.4 a	2.9 ± 0.1 a	2.1 ± 0.4 a	2.5 ± 0.3 a	1.8 ± 0.0 a	2.2 ± 0.1 a
3P4HP	1.9 ± 0.0 a	1.9 ± 0.1 a	1.1 ± 0.1 b	1.2 ± 0.1 b	ND	ND	ND	ND
DTBPHm	41.5 ± 5.0 a	46.0 ± 0.9 a	44.0 ± 0.7 a	47.0 ± 1.0 a	49.2 ± 9.7 a	38.7 ± 4.8 a	39.2 ± 2.6 a	40.8 ± 1.3 a
Coumaran	202.5 ± 4.54 a	196.6 ± 43.0 a	240.6 ± 66.7 a	219.0 ± 3.9 a	1096.6 ± 651.1 a	622.4 ± 312.0 a	970.0 ± 132.30 a	981.9 ± 22.0 a
Ethyl salicylate	6.1 ± 0.97 b	11.8 ± 3.6 a	4.3 ± 0.3 b	4.8 ± 0.3 b	5.8 ± 0.7 b	5.3 ± 0.3 b	5.3 ± 0.1 b	5.6 ± 0.0 b
4-Ethylphenol	1466.5 ± 3.1 b	1480.8 ± 5.3 b	1482.8 ± 14.6 b	1464.6 ± 0.9 b	8167.3 ± 3706.2 a	6012.7 ± 1923.9 ab	11677.1 ± 140.4 a	9968.4 ± 1214.6 a
4-Vinylguaiacol	329.1 ± 9.4 a	310.5 ± 56.6 a	372.2 ± 93.1 a	371.6 ± 4.6 a	791.7 ± 394.5 a	548.7 ± 211.3 a	656.6 ± 15.3 a	617.2 ± 60.1 a
*Total*	*2588.3 ± 23.0 b*	*2570.2 ± 185.4 b*	*2643.6 ± 285.7 b*	*2630.6 ± 23.2 b*	*10511.8 ± 4916.4 ab*	*7594.7 ± 2530.2 ab*	*13943.7 ± 312.4 a*	*12194.2 ± 1375.9 a*
***Esters***								
Ethyl Acetate	74687.2 ± 1416.4 a	78072.4 ± 2474.7 a	75663.3 ± 6509.8 a	72540.3 ± 646.6 a	47864.3 ± 1550.9 b	49750.7 ± 3003.3 b	52201.6 ± 1608.2 b	57245.4 ± 1761.9 b
Ethyl butanoate	306.4 ± 15.3 a	315.7 ± 17.8 a	306.2 ± 23.2 a	294.7 ± 11.6 a	87.4 ± 0.1 b	93.1 ± 7.2 b	39.8 ± 0.7 b	42.4 ± 0.7 b
Ethyl hexanoate	395.7 ± 21.7 a	402.1 ± 20.1 a	378.9 ± 12.10 a	366.3 ± 15.6 a	147.0 ± 0.8 bc	154.5 ± 14.4 b	95.2 ± 3.4 c	99.4 ± 3.0 c
Ethyl 3-hexenoate	2.4 ± 0.2 d	3.0 ± 0.4 d	1.6 ± 1.1 d	2.6 ± 0.0 d	8.5 ± 0.0 ab	9.1 ± 0.3 a	6.8 ± 0.3 c	7.0 ± 0.2 bc
Ethyl enanthate	3.1 ± 0.1 a	3.1 ± 0.1 a	3.0 ± 0.0 a	2.8 ± 0.1 a	1.6 ± 0.0 b	1.6 ± 0.1 b	1.5 ± 0.1 b	1.5 ± 0.1 b
Ethyl lactate	3460.5 ± 645.5 a	3190.9 ± 907.9 a	3265.6 ± 604.4 a	3387.4 ± 131.4 a	4251.8 ± 507.7 a	4276.0 ± 520.7 a	4818.9 ± 994.4 a	4173.8 ± 365.8 a
Ethyl octanoate	931.8 ± 18.3 ab	955.1 ± 6.3 a	858.0 ± 39.6 b	898.5 ± 43.4 ab	506.8 ± 18.8 c	527.3 ± 2.3 c	370.5 ± 1.0 d	403.9 ± 4.5 d
Ethyl mesitylacetate	1.1 ± 0.1 a	1.0 ± 0.0 a	1.0 ± 0.0 a	1.0 ± 0.0 a	0.5 ± 0.0 b	0.5 ± 0.1 b	0.4 ± 0.0 b	0.4 ± 0.0 b
Ethyl nonanoate	4.0 ± 0.2 a	4.3 ± 0.1 a	4.2 ± 0.7 a	3.7 ± 1.1 a	7.5 ± 0.6 a	7.9 ± 2.4 a	7.0 ± 0.6 a	6.1 ± 1.5 a
E2H4MP	16.0 ± 0.59 c	16.1 ± 0.8 c	18.3 ± 2.1 bc	18.1 ± 0.2 bc	27.4 ± 2.1 a	25.0 ± 2.3 a	24.6 ± 1.3 a	24.0 ± 1.3 ab
Ethyl caprate	738.6 ± 0.1 b	746.7 ± 14.1 b	888.1 ± 54.1 a	922.8 ± 46.2 a	620.0 ± 58.9 bc	601.1 ± 4.4 c	426.7 ± 4.2 d	462.0 ± 1.6 d
Ethyl benzoate	1.7 ± 0.1 a	1.8 ± 0.1 a	1.7 ± 0.1 a	1.8 ± 0.1 a	1.7 ± 0.2 a	1.7 ± 0.1 a	1.5 ± 0.1 a	1.6 ± 0.0 a
Ethyl 9-decenoate	17.1 ± 1.1 a	18.4 ± 1.7 a	16.2 ± 2.3 a	18.9 ± 1.3 a	3.9 ± 0.0 b	3.5 ± 0.0 b	3.4 ± 0.1 b	3.5 ± 0.0 b
Ethyl laurate	135.3 ± 2.6 bcd	137.3 ± 4.6 abcd	173.2 ± 13.4 bc	182.6 ± 11.6 a	153.5 ± 26.7 abc	119.4 ± 2.7 cd	96.8 ± 0.0 d	99.3 ± 2.9 d
Ethyl myristate	24.0 ± 0.5 a	23.3 ± 5.7 a	22.9 ± 4.2 a	27.5 ± 1.1 a	22.3 ± 3.9 a	20.8 ± 1.6 a	19.4 ± 2.1 a	24.8 ± 3.9 a
Ethyl hexadecanoate	38.7 ± 1.0 bc	33.6 ± 4.8 c	39.9 ± 1.1 bc	40.0 ± 2.2 bc	60.9 ± 11.8 a	54.9 ± 5.3 ab	61.4 ± 3.3 a	70.4 ± 1.0 a
Isobutyl acetate	206.8 ± 13.2 a	216.3 ± 17.0 a	158.9 ± 14.6 b	154.6 ± 0.4 b	61.8 ± 8.4 c	68.0 ± 12.2 c	17.2 ± 2.2 d	33.4 ± 6.7 cd
Isopentyl acetate	4878.4 ± 338.0 a	5027.3 ± 383.3 a	3790.5 ± 325.2 b	3603.7 ± 160.7 b	2382.1 ± 5.8 c	2515.1 ± 158.2 c	1110.7 ± 11.2 d	1182.4 ± 48.9 d
Hexyl acetate	9.10 ± 0.5 a	9.3 ± 0.3 a	6.8 ± 0.4 b	6.5 ± 0.1 b	1.3 ± 0.1 cd	2.3 ± 0.0 c	1.2 ± 0.7 cd	0.7 ± 0.5 d
Heptyl acetate	0.5 ± 0.3 a	0.4 ± 0.2 a	0.3 ± 0.1 a	0.2 ± 0.0 a	0.2 ± 0.1 a	0.1 ± 0.1 a	0.1 ± 0.2 a	0.2 ± 0.1 a
2-Ethyl-1-hexanol acetate	13.1 ± 1.3 a	13.2 ± 0.5 a	13.0 ± 1.3 a	8.1 ± 6.8 a	8.6 ± 0.3 a	8.3 ± 0.4 a	9.2 ± 0.2 a	9.9 ± 0.7 a
Octyl acetate	7.5 ± 0.5 ab	7.8 ± 0.4 ab	7.2 ± 0.9 ab	8.3 ± 0.9 a	4.5 ± 1.2 b	5.7 ± 0.3 ab	5.4 ± 1.6 ab	5.3 ± 0.6 ab
Trimethylene acetate	129.3 ± 33.4 a	135.0 ± 39.4 a	122.4 ± 33.8 a	105.1 ± 14.0 a	435.3 ± 324.9 a	243.1 ± 123.2 a	265.8 ± 116.0 a	381.8 ± 9.4 a
Isobutyl hexanoate	0.6 ± 0.2 ab	0.5 ± 0.0 a	0.4 ± 0.0 c	0.4 ± 0.0 bc	ND	ND	ND	ND
Methyl octanoate	5.2 ± 0.1 a	5.5 ± 0.1 a	5.1 ± 0.2 a	3.4 ± 2.4 a	2.6 ± 0.1 a	2.7 ± 0.0 a	2.3 ± 0.0 a	2.4 ± 0.0 a
Isopentyl hexanoate	7.0 ± 0.1 a	7.4 ± 0.3 a	6.4 ± 0.6 a	7.0 ± 0.4 a	2.3 ± 0.0 b	2.3 ± 0.1 b	1.5 ± 0.0 b	1.7 ± 0.0 b
Isobutyl octanoate	2.7 ± 0.5 a	2.6 ± 0.6 a	2.3 ± 0.4 a	2.4 ± 0.4 a	3.0 ± 0.4 a	1.6 ± 0.1 a	2.8 ± 1.9 a	4.3 ± 0.1 a
Methyl caprate	12.2 ± 0.2 a	12.1 ± 0.1 a	18.3 ± 1.4 a	18.9 ± 1.2 a	6.8 ± 1.1 a	6.7 ± 0.1 a	3.4 ± 0.1 a	4.5 ± 0.3 a
Isoamyl octanoate	9.4 ± 0.3 ab	9.7 ± 0.4 a	7.7 ± 0.7 b	8.3 ± 0.8 ab	4.5 ± 0.2 c	4.6 ± 0.0 c	3.7 ± 0.1 c	4.2 ± 0.1 c
Diethyl succinate	125.6 ± 2.5 b	126.4 ± 13.2 b	216.0 ± 45.4 a	227.6 ± 5.7 a	100.5 ± 30.0	87.5 ± 13.5 b	108.4 ± 3.5 b	92.0 ± 9.5 b
Isoamyl decanoate	4.6 ± 0.1 a	4.6 ± 0.4 a	5.2 ± 0.0 a	5.5 ± 0.4 a	3.2 ± 0.3 b	2.9 ± 0.0 bc	2.0 ± 0.0 d	2.2 ± 0.0 cd
IMDMMPo	19.7 ± 1.5 a	19.4 ± 1.3 a	12.5 ± 1.0 b	13.3 ± 0.4 b	3.1 ± 0.7 c	2.5 ± 0.9 c	2.0 ± 0.3 c	2.3 ± 0.8 c
NA3MBE	5.2 ± 0.1 a	5.0 ± 0.2 a	5.0 ± 0.4 a	5.3 ± 0.2 a	2.1 ± 0.2 b	1.9 ± 0.0 b	1.9 ± 0.0 b	1.8 ± 0.0 b
*Total*	*86200.3 ± 1208.5 a*	*89527.3 ± 1927.2 a*	*86020.1 ± 6080.8 a*	*82887.4 ± 822.9 a*	*56786.7 ± 2501.9 b*	*58602.3 ± 2531.9 b*	*59713.0 ± 716.2 b*	*64394.2 ± 1452.0 b*
Others								
Styrene	97.1 ± 1.7 ab	97.8 ± 2.1 ab	83.3 ± 3.5 b	86.5 ± 2.7 b	93.5 ± 3.5 ab	105.7 ± 7.5 a	65.5 ± 4.2 c	67.3 ± 1.9 c
α-Ionene	7.8 ± 0.2 a	6.9 ± 0.2 a	6.4 ± 2.2 a	7.2 ± 0.7 a	2.6 ± 0.4 a	2.2 ± 0.1 a	3.8 ± 0.2 a	3.8 ± 0.3 a
ODETMq	2.7 ± 0.1 c	2.8 ± 0.1 c	2.6 ± 0.0 cd	2.7 ± 0.2 c	6.0 ± 0.2 b	6.7 ± 0.0 ab	6.6 ± 0.1 b	7.5 ± 0.5 a
α-Cedrene	2.1 ± 0.0 a	2.0 ± 0.1 a	1.2 ± 0.2 a	1.4 ± 0.2 a	0.2 ± 0.0 b	0.1 ± 0.0 b	0.1 ± 0.0 b	0.1 ± 0.0 b
α-Calacorene	6.2 ± 0.1 a	5.8 ± 0.2 ab	5.2 ± 1.4 ab	5.9 ± 0.5 ab	4.4 ± 0.6 ab	3.9 ± 0.0 b	4.0 ± 0.0 ab	3.9 ± 0.2 b
β-Ionone	7.4 ± 0.8 a	7.3 ± 0.8 a	6.5 ± 0.4 a	6.8 ± 0.2 a	1.7 ± 0.3 c	1.8 ± 0.2 c	4.5 ± 0.0 b	4.1 ± 0.0 b
DMUDL	0.4 ± 0.1 ab	0.4 ± 0.1 ab	0.5 ± 0.0 a	0.5 ± 0.0 a	0.2 ± 0.1 c	0.2 ± 0.0 c	0.3 ± 0.0 bc	0.3 ± 0.0 bc
Cedrol	0.5 ± 0.1 a	0.4 ± 0.0 a	0.3 ± 0.0 b	0.3 ± 0.0 b	ND	ND	ND	ND
*Total*	*124.1 ± 1.0 a*	*123.5 ± 0.8 a*	*106.0 ± 7.77 a*	*111.2 ± 4.5 a*	*108.7 ± 1.9 a*	*120.6 ± 7.2 a*	*84.8 ± 4.0 b*	*86.9 ± 2.8 b*

DF and DP indicate dried goji berry fermented wine made of free-run and pressed juice, respectively. DF-G and DP-G represent dried goji berry fermented wine made of free-run and pressed juice after the glycosidase treatment, respectively. FF and FP are fresh goji berry fermented wine made of free-run and pressed juice, respectively. FF-G and FP-G stand for fresh goji berry fermented wine made of free-run and pressed juice after the glycosidase treatment, respectively. *: Different letters in the same row indicate significant differences at *p* ≤ 0.05. **, ***: “ND” and “TR” represent “not detected” and “trace amount”, respectively. Data were expressed as the mean ± standard deviation.

**Table 4:** T-test on concentration of volatile compounds in wine made of fresh and dried berry fruit and two-factor variance analysis on concentration of volatile compounds in goji berry fermented wine with pressing and glycosidase treatments.

Volatile	T-test between DW and FW	Dried Goji Berry Fermented Wine			Fresh Goji Berry Fermented Wine		
		*p* Value			*p* Value		
		Pressing	Glycosidase	Interaction	Pressing	Glycosidase	Interaction
***Volatile acids***							
DMECA	16.35 ***	0.639	0.316	0.584	0.758	0.386	0.427
Acetic acid	0.33 ***	0.582	0.527	0.890	0.145	0.341	0.706
Butanoic acid	3.70 ***	0.990	0.994	0.924	0.710	0.624	0.651
α-Methylbutyric acid	3.47 ***	0.599	0.537	0.66	0.567	0.367	0.489
Octanoic acid	1.51 **	0.626	0.225	0.784	0.559	0.506	0.732
Decanoic acid	0.98	0.043 *	0.100	0.223	0.047 *	0.663	0.505
*Total*	1.37 *	*0.052*	*0.097*	*0.313*	*0.107*	*0.546*	*0.687*
***Higher alcohols***							
2-Methyl-1-propanol	1.63 ***	0.648	0.498	0.789	0.001 **	0.934	0.829
Isopentyl alcohol	0.96	1.000	0.273	0.720	0.0001 ***	0.619	0.765
Pentyl alcohol	0.59 ***	0.278	0.598	0.164	0.014 *	0.565	0.350
1-Hexanol	0.71 *	0.362	0.487	0.235	0.000 ***	0.007 **	0.673
2-Heptanol	0.81 ***	0.014 *	0.707	0.608	0.314	0.317	0.029 *
1-Octen-3-ol	0.58 **	0.478	0.042 *	0.777	0.557	0.617	0.475
2-Ethyl-1-hexanol	0.30 **	0.002 **	0.949	0.146	0.000 ***	0.633	0.261
[*R*-(R*,R*)]-2,3-Butanediol	0.24 **	0.655	0.746	0.821	0.001 ***	0.801	0.423
1-Octanol	0.46 **	0.458	0.754	0.832	0.000 ***	0.408	0.125
[*S*-(R*,R*)]-2,3-Butanediol	0.43 **	0.638	0.624	0.498	**0.827**	0.929	0.290
trans-2-Octenol	0.56 ***	0.543	0.240	0.481	**0.085**	0.879	0.227
1-Nonanol	0.43 ***	0.010 **	0.000 ***	0.040 *	0.005 **	0.300	0.278
Methionol	1.73 ***	0.855	0.724	0.711	**0.601**	0.587	0.713
(*E*)-5-Decen-1-ol	0.00 **	0	0	0	0.000 ***	0.077	0.384
Phenylethyl Alcohol	1.49 **	0.874	0.939	0.872	0.972	0.52	0.718
*Total*	0.36 **	*0.659*	*0.704*	*0.750*	*0.877*	*0.833*	*0.405*
***Aldehyde/Ketones***							
Furfural	39.11 ***	0.103	0.59	0.369	0.733	0.782	0.801
Decanal	1.26	0.176	0.029 **	0.453	0.859	0.795	0.070
Safranal	3.85 ***	0.111	0.728	0.810	0.000 ***	0.176	0.270
2-Butyl-2-octenal	1.25 ***	0.649	0.761	0.963	**0.811**	0.598	0.639
Isobutyl ketone	16.21 **	0.682	0.088	0.126	0.003 **	0.003 **	0.378
TMCHN	0.00 *	0	0	0	0.002 **	0.463	0.463
6M5H2NE	***	0.102	0.821	0.745	0	0	0
2-Undecanone	2.86 ***	0.001 ***	0.774	0.598	0.002 **	0.281	0.222
β-Cyclocitral	1.46 ***	0.003 **	0.977	0.855	0.000 ***	0.836	0.055
Dihydropseudoionone	2.38 ***	0.911	0.342	0.308	0.000 ***	0.260	0.167
2-Acetylpyrrole	***	0.822	0.878	0.956	0	0	0
TMHAAL	5.22 ***	0.916	0.426	0.619	0.851	0.396	0.043 *
*Total*	16.03 ***	*0.065*	*0.191*	*0.182*	*0.879*	*0.812*	*0.259*
***Benzene***							
Isodurene	3.86 ***	0.125	0.555	0.568	0.001 **	0.221	0.820
TEMLNE	2.16 ***	0.858	0.839	0.238	**0.052**	0.158	0.896
DPPXL	1.62 ***	0.258	0.711	0.352	0.044 *	0.776	0.950
Naphthalene	2.20 ***	0.193	0.063	0.154	**0.137**	0.416	0.439
TMDHPE	2.78 ***	0.355	0.851	0.417	0.007 **	0.341	0.553
M2PB	0.83 ***	0.019 **	0.842	0.024 **	0.629	0.522	0.420
Phenethyl acetate	3.86 ***	0.055	0.857	0.577	0.008 **	0.228	0.461
β-Methylnaphthalene	3.25 ***	0.882	0.903	0.269	0.029 *	0.583	0.619
Benzyl alcohol	0.75	0.884	0.989	0.840	0.027 *	0.778	0.926
Ethyl dihydrocinnamate	0.41 ***	0.677	0.600	0.821	0.477	0.223	0.498
α-Methylnaphthalene	5.12 ***	0.753	0.858	0.376	0.097	0.179	0.286
4-Tolylcarbinol	1.59 **	0.650	0.549	0.884	0.441	0.580	0.504
3P4HP	***	0.001 **	0.179	0.498	0	0	0
Coumaran	0.23 ***	0.342	0.651	0.793	0.677	0.424	0.402
4-Ethylphenol	0.16 ***	0.996	0.745	0.044 *	0.072	0.278	0.892
4-Vinylguaiacol	0.53 ***	0.249	0.816	0.828	0.845	0.427	0.558
*Total*	0.25 ***	*0.738*	*0.900*	*0.980*	*0.117*	*0.312*	*0.787*
***Esters***							
Ethyl Acetate	1.45 ***	0.418	0.961	0.267	0.016 *	0.077	0.341
Ethyl butanoate	5.41 ***	0.438	0.935	0.445	0.167	0.290	0.346
Ethyl hexanoate	3.11 ***	0.104	0.818	0.494	0.001 ***	0.335	0.768
Ethyl 3-hexenoate	0.31 ***	0.196	0.133	0.654	0.000 **	0.059	0.330
Ethyl enanthate	1.94 ***	0.058	0.156	0.148	0.013 **	0.772	0.402
Ethyl lactate	0.76 **	0.999	0.878	0.686	**0.636**	0.532	0.502
Ethyl octanoate	2.01 ***	0.041 *	0.218	0.714	0.000 ***	0.017 **	0.404
Ethyl mesitylacetate	2.42 ***	0.229	0.444	0.038*	0.089	0.773	0.788
Ethyl nonanoate	0.56 ***	0.682	0.810	0.441	0.337	0.800	0.576
E2H4MP	0.68 ***	0.057	0.980	0.832	0.209	0.299	0.516
Ethyl caprate	1.56 ***	0.003 **	0.451	0.632	0.001 **	0.717	0.266
Ethyl benzoate	1.08 *	0.432	0.183	0.859	0.037 *	0.509	0.491
Ethyl 9-decenoate	4.92 ***	0.907	0.170	0.586	0.009 **	0.024 *	0.004 **
Ethyl laurate	1.34 **	0.003 **	0.433	0.605	0.016 *	0.172	0.128
Ethyl hexadecanoate	0.61 ***	0.123	0.266	0.247	**0.165**	0.764	0.190
Isobutyl acetate	4.09 ***	0.004 **	0.790	0.497	0.002 **	0.126	0.439
Isopentyl acetate	2.41 ***	0.005 **	0.936	0.491	0.000 ***	0.156	0.629
Hexyl acetate	5.82 ***	0.000 ***	0.829	0.342	0.054	0.466	0.058
Heptyl acetate	2.31 *	0.283	0.487	0.989	**0.658**	0.983	0.454
2-Ethyl-1-hexanol acetate	1.32 *	0.351	0.379	0.370	0.023 **	0.596	0.155
Octyl acetate	1.47 ***	0.831	0.236	0.540	0.739	0.534	0.416
Trimethylene acetate	0.37 **	0.457	0.811	0.634	0.911	0.783	0.300
Isobutyl hexanoate	7.32 ***	0.004 **	0.095	0.639	0.000 ***	0.476	0.476
Methyl octanoate	1.94 **	0.256	0.439	0.327	0.000 ***	0.033 **	0.940
Isopentyl hexanoate	3.60 ***	0.149	0.162	0.722	0.000 ***	0.085	0.028 **
Methyl caprate	0.81	0.000 ***	0.106	0.253	0.688	0.144	0.346
Isoamyl octanoate	2.06 ***	0.022 *	0.348	0.765	0.003	0.022	0.076
Diethyl succinate	1.79 **	0.005 **	0.729	0.764	0.635	0.292	0.892
Isoamyl decanoate	1.95 ***	0.027 **	0.469	0.55	0.001 **	0.950	0.143
IMDMMP	6.60 ***	0.001 **	0.798	0.499	0.287	0.868	0.406
NA3MBE	2.67 ***	0.756	0.954	0.255	0.059	0.262	0.456
*Total*	1.44 ***	*0.222*	*0.965*	*0.234*	*0.034*	*0.086*	*0.387*
***Others***							
Styrene	1.10	0.002 **	0.355	0.527	0.000 ***	0.105	0.198
α-Ionene	2.48 ***	0.783	0.169	0.701	**0.078**	0.212	0.988
ODETM	0.40 ***	0.407	0.185	0.865	0.023 *	0.012 *	0.622
α-Cedrene	1.92 ***	0.772	0.277	0.772	**0.446**	0.333	0.142
α-Calacorene	1.42 ***	0.435	0.787	0.372	**0.418**	0.232	0.454
β-Ionone	2.32 ***	0.158	0.788	0.666	0.000 ***	0.135	0.131
DMUDL	1.85 ***	0.024 *	0.915	0.928	0.152	0.442	0.702
Cedrol	***	0.002 **	0.808	0.654	0	0	0
*Total*	1.18 *	0.011 *	*0.414*	*0.465*	0.001 **	*0.123*	*0.288*

The result of T-test between DW and DF is calculated using the volatile concentration in wine made of dried and fresh goji berry and presented with the concentration ratio. *p* value of pressing effect is calculated using the volatile concentration in wine made of free-run and pressed juice. *p* value of glycosidase effect is calculated using the volatile concentration in wine with and without glycosidase treatment. Interaction represent the interactive effect made of pressing and glycosidase treatments on volatile concentration in wine. *, **, and *** indicate the significance effect at *p* ≤ 0.05, 0.01, and 0.001, respectively.

**Table 5:** Odor activity value (OAVs) of volatile compound in goji berry fermented wines.

Volatile	Threshold (μg/L)	Aroma Descriptor	Aroma Series	DF	DF-G	DP	DP-G	FF	FF-G	FP	FP-G
Butanoic acid	173 [45]	Butter, cheese, stinky [45]	1 [46]	8.7 a	8.6 a	8.6 a	8.7 a	2.4 b	2.4 b	2.4 b	2.2 b
Octanoic acid	500 [37]	Rancid, cheese, fatty acid [28]	1 [28]	2.0 a	1.6 a	2.1 a	1.8 a	1.5 a	1.2 a	1.2 a	1.1 a
Decanoic acid	1000 [37]	Fatty, rancid [37]	1 [46]	2.7 a	2.2 a	6.1 a	3.3 a	5.0 a	4.2 a	2.6 a	2.8 a
Isopentyl alcohol	30000 [47]	Solvent, sweety, alcohol, polish [38]	1,2 [38]	6.3 b	6.2 b	6.3 b	6.1 b	7.2 a	7.2 a	5.8 b	5.8 b
[*R*-(R*,R*)]-2,3-Butanediol	150000 [28]	Fruit, sweet, butter [28]	1,2,3 [28]	2.5 b	2.4 b	2.3 b	1.9 b	11.8 a	6.5 a	8.8 a	11.6 a
[*S*-(R*,R*)]-2,3-Butanediol	150000 [28]	Fruit, sweet, butter [28]	1,2,3 [28]	0.8 a	0.8 a	0.8 a	0.6 a	2.0 a	1.3 a	1.4 a	2.3 a
Methionol	1000 [28]	Cabbage, cooked potato, garlic [28]	4 [28]	1.3 a	1.2 a	1.3 a	1.3 a	0.8 a	0.5 a	0.8 a	0.8 a
Phenylethyl Alcohol	14000 [28]	Roses, honey [28]	5 [28]	1.5 a	1.4 a	1.4 a	1.4 a	1.1 a	0.8 a	1.0 a	0.9 a
Ethyl cinnamate	1.1 [47]	Flowery, balsamic [48]	5 [46]	1.7 a	2.3 a	2.2 a	2.7 a	1.9 a	2.3 a	1.6 a	2.0 a
4-Ethylphenol	440 [28]	Phenolic, leather [28]	6 [28]	3.3 b	3.4 b	3.4 b	3.3 b	18.6 a	13.7 ab	26.5 a	22.7 a
Ethyl Acetate	5000 [8,49]	Spicy, pineapple, fruity [38]	3 [46]	6.2 a	6.5 a	6.3 a	6.0 a	4.0 b	4.1 b	4.4 b	4.8 b
Ethyl hexanoate	14 [47]	Banana, green, apple [28]	3 [28]	4.9 a	5.0 a	4.7 a	4.6 a	1.8 bc	1.9 b	1.2 c	1.2 c
Ethyl octanoate	5 [28]	Sweet, floral, fruity, banana, pear [28]	3,5 [46]	1.6 ab	1.6 a	1.5 b	1.5 ab	0.9 c	0.9 c	0.6 d	0.7 d
Ethyl caprate	200 [37]	Brandy, fruity, grape [37]	3 [37]	3.7 b	3.7 b	4.4 a	4.6 a	3.1 bc	3.0 c	2.1 d	2.3 d
Isopentyl acetate	30 [47]	Sweety, fruity [38]	2,3 [46]	30.5 a	31.4 a	23.7 b	22.5 b	14.9 c	15.7 c	6.9 d	7.4 d
β-Ionone	0.09 [47]	Violet [32]	5 [46]	92.6 a	91.6 a	80.7 a	84.8	21.8 c	21.8 c	56.8 b	50.6 b

DF and DP indicate dried goji berry fermented wine made of free-run and pressed juice, respectively. DF-G and DP-G represent dried goji berry fermented wine made of free-run and pressed juice after the glycosidase treatment, respectively. FF and FP are fresh goji berry fermented wine made of free-run and pressed juice, respectively. FF-G and FP-G stand for fresh goji berry fermented wine made of free-run and pressed juice after the glycosidase treatment, respectively. Volatile compounds with available odor threshold and aroma descriptor from literature and OAV above 1 are listed. 1, 2, 3, 4, 5, and 6 in aroma series column represent fatty, caramel, fruity, herbaceous (or vegetal), floral, and chemical note, respectively.
